# Superior Mesenteric Artery Syndrome: Could it Involve a Potential Familial Pattern?

**DOI:** 10.7759/cureus.15072

**Published:** 2021-05-17

**Authors:** Kalvin Zee, Michael Stephens, Matthew Fabiszak

**Affiliations:** 1 Internal Medicine, Rocky Vista University College of Osteopathic Medicine, Ivins, USA; 2 Internal Medicine, Intermountain Healthcare, St. George, USA

**Keywords:** superior mesenteric artery syndrome, duodenal obstruction, intestinal obstruction, small bowel obstruction, superior mesenteric artery

## Abstract

Superior mesenteric artery (SMA) syndrome often presents with small bowel obstruction due to acute angulation of the SMA, thereby compressing the duodenum. This syndrome is a rare, sporadic disease, which is often caused by rapid weight loss. However, there may be a genetic predisposition to SMA syndrome, due to a congenitally shortened ligament of Treitz or a more distal origination of the SMA on the abdominal aorta. In this study, we present a patient who was diagnosed with SMA syndrome despite not exhibiting the classic weight-loss clinical picture. Interestingly, the patient reported a family history of SMA syndrome in her mother who had experienced a similar presentation.

## Introduction

Superior mesenteric artery (SMA) syndrome is a rare cause of upper gastrointestinal obstruction that affects an estimated 0.1-3% of people in the United States [1]. The obstruction occurs when the acute angulation of the SMA causes compression of the third part of the duodenum between the SMA and the aorta, leading to a small bowel obstruction [2]. Symptoms range from postprandial nausea and bilious vomiting to abdominal pain and weight loss, and the severity of symptoms depends on the degree of compression [2]. Initial treatment of SMA syndrome is conservative, which consists of fluid resuscitation, total parenteral nutrition, and gastric decompression [3]. Nutritional support is essential and usually performed by providing small, frequent meals in order to increase the mesenteric fat pad, thereby relieving the obstruction [3]. Surgical intervention is required when conservative therapy fails, and the standard operative procedure is laparoscopic duodenojejunostomy [3,4]. This operation bypasses the segment of the duodenum that is compressed, creating a connection between the proximal duodenum and jejunum. Although generally sporadic, some researchers have speculated that there is a genetic predisposition to SMA syndrome, with individuals born with a congenitally shortened ligament of Treitz being susceptible to the resultant obstruction by the SMA [5]. In this paper, we describe a case of SMA syndrome in a female who had developed duodenal obstruction due to the condition. Her family medical history was significant with her mother reportedly being diagnosed with SMA syndrome at a similar age. However, neither woman showed the classic weight-loss presentation associated with the syndrome, which raised the suspicion regarding a genetic predisposition to SMA syndrome.

## Case presentation

The patient was a 39-year-old female with a past medical history of pulmonary embolism, anxiety, chronic nausea, constipation, hypothyroidism, and chronic pain who presented with two days of acute epigastric abdominal pain with radiation to the lower quadrants in the setting of chronic abdominal discomfort of approximately one year's duration. She also had secondary complaints of nausea and vomiting. Her chronic pain had affected her food intake, and she reported losing 15 pounds in the previous two weeks and an estimated 90 pounds over the past year due to her pain. She reported having a normal bowel movement the day before the presentation.

The patient had a family history of SMA syndrome in her mother, who stated that she had not had any weight loss prior to the appearance of her symptoms and diagnosis. The patient had a surgical history of cholecystectomy and hysterectomy. Her medications included those for thyroid replacement, as well as bupropion, buprenorphine, gabapentin, oxcarbazepine, omeprazole, fluoxetine, trazodone, and rivaroxaban. She reported allergies to penicillin, naproxen sodium, meloxicam, aspirin, and ibuprofen.

**Figure 1:**
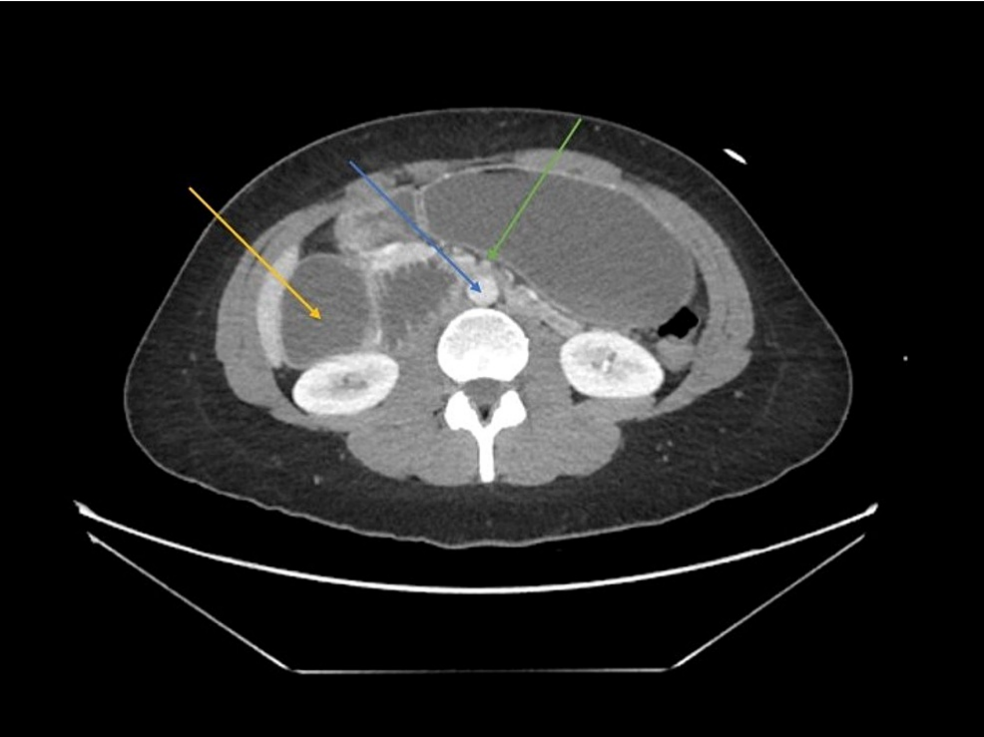
CT scan of the patient The SMA (right, green arrow) can be visualized anterior to the aorta (middle, blue arrow) with dilated proximal duodenum (left, yellow arrow) CT: computed tomography; SMA: superior mesenteric artery

On examination, her vitals were found to be normal. The patient was very anxious and emotional, and her abdomen was mildly distended and tender to palpation in all quadrants. Bowel sounds were present. A complete blood count (CBC) and basic metabolic panel (BMP) were performed, which showed mild hyponatremia, hypochloremia, and hyperglycemia. The patient had normal lactic acid and lipase levels. A CT scan of her abdomen was performed, which revealed a severely distended small bowel and dilated duodenal C-loop up to the level where the duodenum crosses between the aorta and SMA (Figure 1). A nasogastric tube was placed for decompression and IV fluids were given, which provided minimal relief. The patient was also started on her home medications as well as acetaminophen, Flexeril, Miralax, and Phenergan, which helped to alleviate her nausea.

Three days after the admission to the hospital, the patient underwent a laparoscopic Roux-en-Y duodenojejunostomy without complications. She subsequently suffered from postoperative ileus, but it resolved soon and she was able to tolerate a full liquid diet before being discharged on postoperative day three.

## Discussion

SMA syndrome is commonly caused by a depletion/loss of the fat pad enveloping the SMA and the third part of the duodenum resulting in a constriction of the duodenum between the SMA and the aorta [2]. It is usually associated with a history of weight loss including conditions such as malignancy, malabsorption syndromes, and bariatric surgery [1]. Patients with SMA syndrome usually present with symptoms of small bowel obstruction such as abdominal distention, abdominal pain and cramping, halitosis, constipation, diarrhea, inability to pass flatus, nausea and emesis of partially digested food or bile, early satiety, and weight loss [2]. Patients usually begin to experience symptoms when the angle between the aorta and the SMA is reduced below 25º [3]. The normal range is 38-65º [3]. Congenital SMA syndrome also occurs when this angle is reduced, but not due to a reduction of the fat pad. It can occur if the ligament of Treitz is shortened, raising the third part of the duodenum superiorly and pulling it into the aortomesenteric angle [5]. This can also occur when the SMA originates lower than normal off the aorta, thereby pulling the aortomesenteric angle towards the duodenum [6].

The treatment of SMA syndrome includes conservative medical management initially with fluid resuscitation, electrolytes, total parenteral nutrition, and gastric decompression with a nasogastric tube [3]. If the conservative approach fails, surgical intervention is required, with the standard operative treatment being laparoscopic duodenojejunostomy to bypass the obstructed segment of the duodenum [3].

Our patient presented with nonspecific severe epigastric pain, emesis, inability to tolerate food, and a history of weight loss of 90 lbs after the onset of pain. The diagnosis of small bowel obstruction due to SMA syndrome was confirmed via a CT scan. Although she had a history of significant weight loss, her epigastric pain had preceded the onset of weight loss, and the patient stated that she had lost weight due to her postprandial pain. Her family medical history included her mother receiving a similar diagnosis at age 40, which raised the suspicion for a genetic predisposition to SMA syndrome. SMA syndrome is currently not considered to be an inherited condition [7]. However, the condition has been observed in twin studies, indicating the possibility of a familial inheritance pattern in SMA syndrome [8]. Further studies should be performed to correlate the condition with a congenitally shortened ligament of Treitz or lower origination point of the SMA from the aorta.

This study has some limitations. Primarily, we did not evaluate the patient's mother. Although she reported a family history of SMA syndrome, we could not personally verify this information as her medical chart was unavailable. Thus, we were unable to independently verify her family history.

## Conclusions

SMA syndrome is a rare disease that often presents with small bowel obstruction following significant weight loss. Congenital SMA syndrome may be considered in patients who have SMA syndrome without previous weight loss and a reported positive family history of atypically presenting SMA syndrome. Further studies are required to gain more insight into the possible genetic components involved in congenital SMA syndrome.
